# A Thin C-Band Polarization and Incidence Angle-Insensitive Metamaterial Perfect Absorber

**DOI:** 10.3390/ma8041666

**Published:** 2015-04-13

**Authors:** Humberto Fernández Álvarez, Maria Elena de Cos Gómez, Fernando Las-Heras

**Affiliations:** Signal Theory and Communications Area, Department of Electrical Engineering, University of Oviedo, Multi-Purpose Building, Module 8, Gijón 33203, Asturias, Spain; E-Mails: medecos@uniovi.es (M.E.C.G.); flasheras@uniovi.es (F.L.-H.)

**Keywords:** metamaterial absorber (MMA), double-negative media (DNM), negative refractive index (NIR), left-handed media (LHM), polarization insensitive, angular stability

## Abstract

A novel metamaterial absorber design able to operate in the C frequency band is presented, along with an analysis and a method to improve both its bandwidth and its angular stability. Simulation results for a FR4-based design are shown for comparison with existing designs. In addition, a simplified equivalent circuit is provided for a better understanding of the great angular stability and wide bandwidth exhibited by the proposed structure. Moreover, simulations, manufacturing and measurements of a thinner and more flexible metamaterial absorber, keeping the angular stability of the former one, while providing a wide bandwidth, are presented.

## 1. Introduction

Metamaterial absorbers (MMA) have been attracting attention in recent years [1,2,3,4,5,6,7,8,9,10,11] due to their capabilities of almost perfectly absorbing electromagnetic radiation through low profile periodic structures. MMA are generally implemented using metasurfaces exhibiting LHM behavior [12], and they can be described by their constitutive parameters [13,14,15,16].

A metamaterial can be described as an effective medium [5,7,10,15,16,17], with a complex electric permittivity ε(*w*) *=* ε_1_(*w*) *+ j*ε_2_(*w*) and a complex electric permeability *µ*(*w*) *= µ*_1_(*w*) *+ jµ*_2_(*w*). Both ε_2_ and *µ*_2_ are directly related to the material losses, which are the key issue for achieving absorption in an MMA. Another important consideration is the need for impedance matching to free space to obtain zero reflection. This can be achieved by matching the permeability and the permittivity ($Z=\sqrt{\frac{\mu (w)}{\varepsilon (w)}}$) of the MMA.

Some of the MMA applications are antenna behavior improvement, solar cells, cloaking, RCS reduction and shielding [8,9].

Most of the MMA found in the literature are based on square or rectangular unit-cells with square-shaped metallization geometries [1,2,3,5,6,7,9,10,14]. In this contribution, a novel hexagonal-based unit-cell metallization geometry is presented aiming to improve both the angular stability under normal and oblique incidence, while exhibiting a wide bandwidth using a very thin and object shape-adapted structure.

A basic design is firstly presented and then upgraded to improve both the bandwidth and the angular stability, while preserving the size and thickness. Once the final design is reached, it is adjusted to be fabricated in a more flexible and thinner dielectric, and it is measured in an anechoic chamber. Finally, some conclusions are presented.

## 2. Results and Discussion

### 2.1. Metamaterial Absorber Design

**Figure 1:**
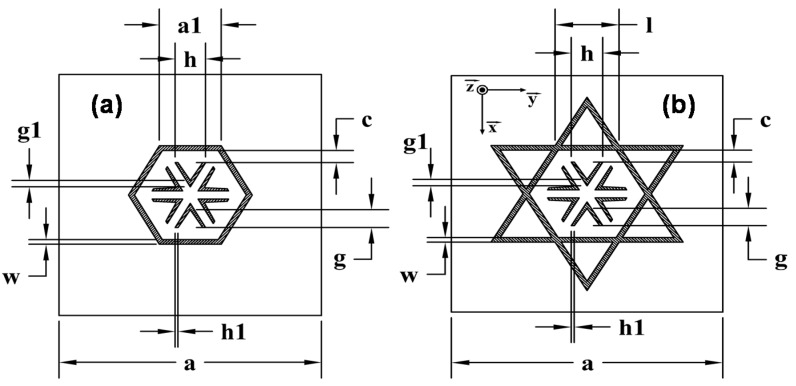
Unit-cell geometries: (**a**) hexagon and (**b**) star.

The absorbing properties of an MMA depend on the electromagnetic characteristics of the dielectric substrate along with the metallization geometry of the unit-cell and its periodicity. FR4 with a relative permittivity ε_r_ = 4.1, a thickness *d* = 1 mm and a loss tangent tanδ = 0.025 is chosen as the dielectric substrate for the starting design, since it is widely used by other authors in previous works [1,2,4,5,7,8,17]. Once the dielectric is fixed, the attention is focused on the metallization geometry of the top face and the periodicity, since, keeping in mind the intended applications, the opposite face of the dielectric spacer is covered by a full metallic ground plane. Copper with a 35-µm thickness and conductivity σ = 5.8 × 10^7^ S/m is used for the metallic parts. Figure 1a shows the basic unit-cell metallization geometry, a regular hexagonal loop with six Λ-shaped inclusions.

Full-wave electromagnetic simulations using the finite element method (FEM)-based commercial software HFSS along with the Bloch-Floquet theory are carried out for the design. A single unit-cell with proper periodic boundary conditions (PBCs) resembling an infinite structure and a port positioned a half-wavelength above it are used to simulate the complex frequency-dependent S-parameters for a uniform incident plane wave. The absorption can be calculated in terms of the frequency-dependent S-parameters [4,14] using Expression (1), where A is the absorption, $\text{R}={\left|{\text{S}}_{11}\right|}^{2}$ is the reflectance and $\text{T}={\left|{\text{S}}_{21}\right|}^{2}$ is the transmittance of the structure.

(1) $$\text{A}=\text{1}-\text{R}-\text{T}=\text{1}-{\left|{\text{S}}_{\text{11}}\right|}^{2}-{\left|{\text{S}}_{\text{21}}\right|}^{2}$$

In this work, *T = 0* due to the backing metallic plate, and thus, the design process aims to reduce the reflectance (*R*) by properly matching the MMA impedance to the free space.

For the starting dimensions—*a* = 16.38 mm, *h* = 1.9 mm, *a1* = 3.86 mm, *g* = 1.21 mm, *g1* = 0.44 mm, *c =* 0.82 mm and *w =* 0.32 mm—the dimension g1 is the difference in the height between the inner and outer triangles of the Λ-shaped metallic strips. The bandwidth obtained in the simulation as the full width at half maximum peak (FWHM), ≈4% for a 96% peak absorption at 8.15 GHz, is narrower than other analyzed designs [2,17] in spite of its potential angular stability due to its six-fold rotational symmetry [18]. To overcome this problem, Λ-shaped metallic strips are added to the outer sides of the hexagon, increasing its inductive behavior and, hence, the bandwidth [19], so that the resulting geometry becomes a star or regular hexagram, preserving the inner Λ-shaped metallic inclusions (see Figure 1b) with dimensions: *a =* 16.38 mm, *l =* 3.86 mm, *h =* 1.9 mm, *h1 =* 0.18 mm, *g =* 1.21 mm, *g1 =* 0.44 mm, *c =* 0.82 mm and *w =* 0.32 mm. It is important to point out that the unit-cell size is preserved.

**Figure 2:**
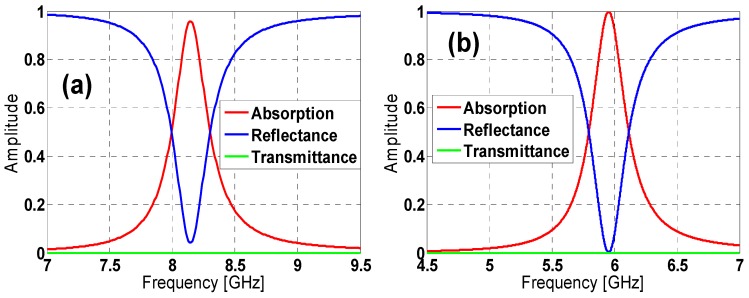
Absorption, reflection and transmission: (**a**) hexagon and (**b**) star design.

The aforementioned results regarding absorption, along with the reflectance and transmittance, under a normal TE-polarized incident plane-wave, for both hexagon and starting unit-cell geometries, are shown in Figure 2.

Table 1 shows the hexagon and star designs’ behavior regarding the maximum absorption peak and the FWHM bandwidth. It is clear that the star design outperforms the hexagon one, not only concerning the peak absorption, but also regarding the bandwidth at a lower resonance frequency (f_r_) (which is more difficult owing to the quality factor corresponding to this kind of absorber exhibiting an LC-parallel resonator-like behavior (see Section 2.2) [3,6,15,16]) and preserving the unit-cell size, achieving a miniaturization MMA.

**Table 1:** Comparative results between hexagonal and star designs.

Prototype	Polarization	Resonance frequency (f_r_) (GHz)	Absorption (%)	Bandwidth (%)
Hexagon	TE	8.146	95.81	3.95
Hexagon	TM	8.191	96.03	3.97
Star	TE	5.631	99.97	5.02
Star	TM	5.638	99.97	5.01

**Figure 3:**
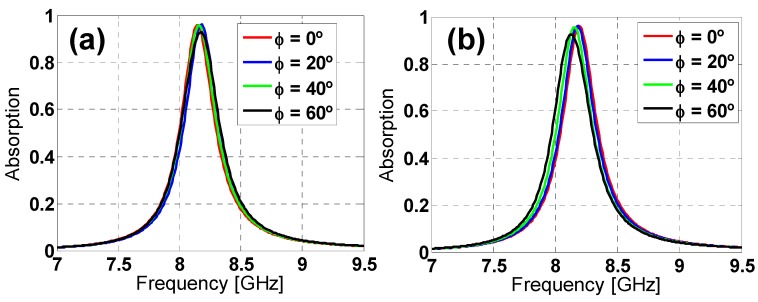
Hexagon design absorption simulation results for different polarization angles (ϕ) of the incident field for both (**a**) TE and (**b**) TM polarizations.

**Figure 4:**
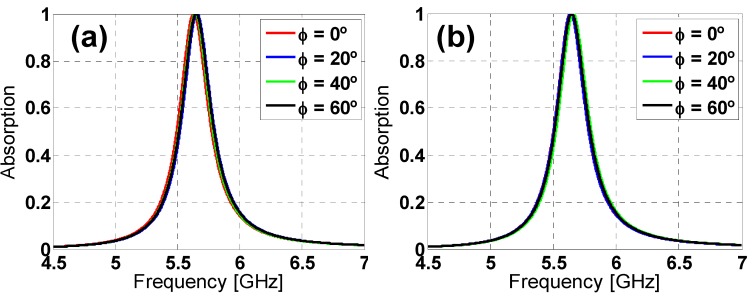
Star design absorption simulation results for different polarization angles (ϕ) of the incident field for both (**a**) TE and (**b**) TM polarizations.

To study the angular stability of both unit-cells under normal incidence, the polarization angle of the incident field (ϕ) has been varied in simulation from 0° to 60° in steps of 20°, for both TE and TM polarized incident plane-waves (considering the electric field along the *y* direction and *x* direction for TE and TM polarization, respectively). From the results depicted in Figure 3 and Figure 4, it can be observed that both designs can be considered stable, especially for TE polarization, due to their six-fold rotational symmetry. However, for TM polarization, the star design is more stable than the hexagon one due to its greater inductive behavior [20].

**Figure 5:**
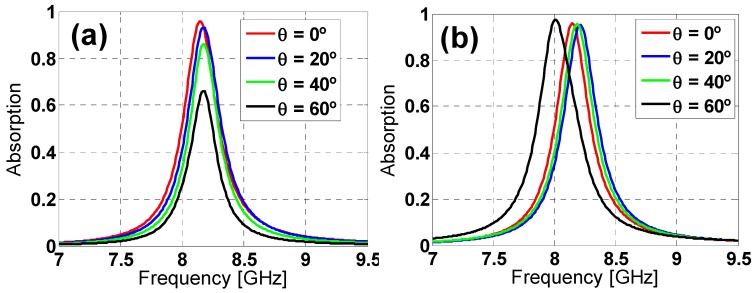
Hexagon design absorption simulation results under different incident angles (θ) for both (**a**) TE and (**b**) TM polarization.

The incidence angle (θ) has been varied from 0° to 60° in steps of 20° to study the angular stability under oblique incidence. From Figure 5, it is easy to notice that the absorption decreases considerably when increasing the incidence angle for the hexagon design under TE polarization, whereas the reduction is smaller for TM polarization, although the resonance frequency shifts rather considerably for θ = 60°.

Compared to a simple hexagonal loop geometry, the inclusion of the inner Λ-shaped metallic strips has the following effects: The angular stability regarding the ϕ polarization angle is improved for both TE and TM polarizations and under oblique incidence is improved from θ = 20°, increasing the absorbance, while almost preserving the resonance frequency (only a 3-MHz shift), although at the expense of a slight reduction in bandwidth (82 MHz (1.4%) for TE and 29 MHz (0.5%) for TM polarization).

**Figure 6:**
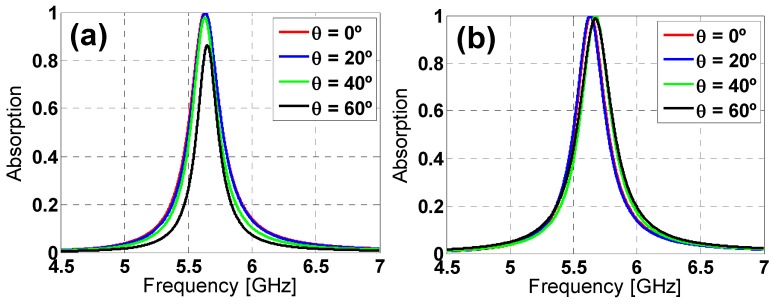
Starting design absorption simulation results under different incident angles (θ) for both (**a**) TE and (**b**) TM polarizations.

Regarding the star design (see Figure 6) under TE polarization, the absorption is almost preserved when increasing the incidence angle, with just a small reduction for θ = 60°, but still 20% higher than for the hexagon one. Once again, for TM polarization, the star design is much more stable than the hexagon one, with no frequency shift, since the added inductive elements compensate somehow the angular dependence of the metallic-backed dielectric slab [20] (which is equivalent to an angularly variant inductance for a reduced thickness of the substrate). Taking all of these results into account, it can be concluded that the star design outperforms the hexagon one, also concerning the angular stability. This conclusion could be explained in terms of the effective inductance of both prototypes, which is clearly greater for the start prototype [20], owing to the additional inductive strips introduced by the tips of the star.

**Figure 7:**
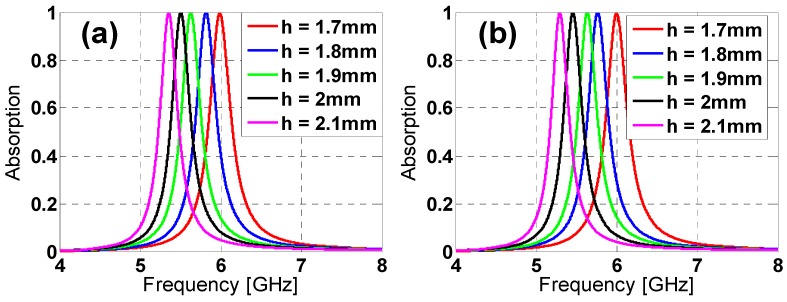
Star design absorption results in the simulation for different lengths of *h* for both (**a**) TE and (**b**) TM polarizations.

**Figure 8:**
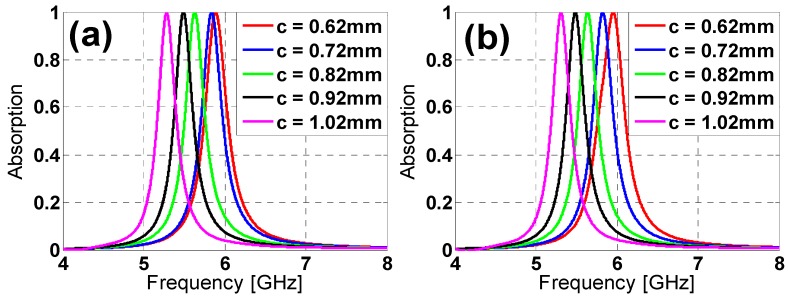
Star design absorption results in the simulation for different sizes of *c* for both (**a**) TE and (**b**) TM polarizations.

**Figure 9:**
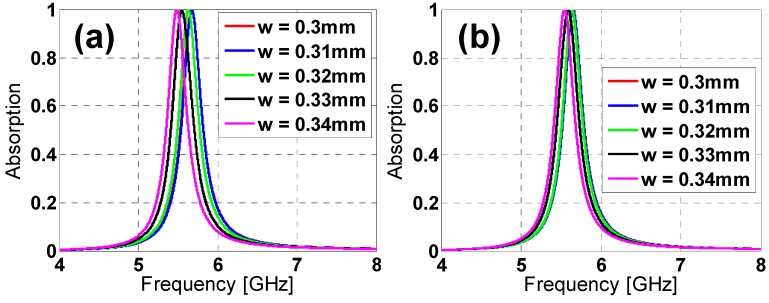
Star design absorption results in the simulation for different lengths of *w* for both (**a**) TE and (**b**) TM polarizations.

Provided the advantages of the star design over the hexagon one, a parametric analysis is presented in order to better understand its performance. The modification of its parameters’ values produces variations in the resonance frequency in the absorption peak and the bandwidth (see Figure 7 and Figure 8). From Figure 7a, the length increase in *h* produces a downwards frequency shift and a bandwidth reduction for both TE and TM polarizations. The same behavior is observed in Figure 9 for an increase in the *c* gap size and the *w* strip width (Figure 9). Thus, from Figure 7 and Figure 8, it is clear that the star design is flexible regarding either the fine or the rough adjustment of the resonance frequency through combinations of the *h*, *c* and *w* parameters’ values.

This observed behavior comes from its metasurface nature, since metamaterials achieve their electromagnetic properties from their geometry and their periodicity, rather than common materials that reach their performance mostly from their chemical or band structure. For these kinds of materials, it can be stated that their wavelength is approximately proportional to their “unwound” copper length L (λ_0_ ~ 2L) [6]. Therefore, an increase in the metamaterial unit-cell length produces an increase in the wavelength and, consequently, a reduction in the resonance frequency.

Most of the MMA previously reported in the literature work at THz frequencies [10,21], where it is relatively easier to obtain absorption due to the high losses of the dielectric substrates (and conductors used in the geometry) and, above all, wherein it is much easier to obtain large bandwidths. The difficulty at these frequencies relies on the fabrication, as well as the availability of instrumentation for measuring the prototypes. However, obtaining a broadband and angularly stable absorber at microwave frequencies, using the same dielectric substrate with the same thickness, is much more challenging, since the capacitive geometry elements generally introduced to decrease the operating frequency, along with the gap reduction between unit-cells, lead to bandwidth reduction and worsen the angular stability.

Compared to some of the most recent MMA presented in the literature, at microwave frequencies, using an identical dielectric substrate with roughly the same thickness [1,5,8,17], it is necessary to point out not only the good performance of the novel one presented in this work regarding the unit-cell bandwidth, but also the achievements concerning its angular behavior. In [1], a metamaterial perfect absorber design is presented with a peak absorption of 99% at 11.65 GHz. The bandwidth at FWHM is 4%, which is 1% lower than the one obtained with the structure presented in this paper. An analysis of its angular stability is carried out, and the results show a sharply deep absorption peak as the angle of incidence increases (at 16°, there is only an absorption of 50%).

The higher the resonance frequency, the easier it is to enlarge the bandwidth (this statement will be clarified in Section 2.2). For example, in [2], the bandwidth is 5.87%, which is slightly greater than the bandwidth at FWHM presented here. However, it is important to realize that the resonance frequency is double the one of this paper (5.631 GHz *vs.* 10.31 GHz [2]). Peak absorption of 95.81% at 10.31 GHz is achieved. Moreover, an angular stability analysis is performed, and the results show a reduction in the bandwidth for both the TE and TM configurations. Nevertheless, in this document, this bandwidth reduction only takes place for the TE configuration. The MMA presented in this paper was scaled by a 0.55 factor in both the x and y directions, along with slight adjustments in c, w and the total thickness, respectively, to c = 0.52 mm, w = 0.28 mm and 0.7 mm, so that the MMA resonates at the same frequency as [2] for a fairer comparison. The absorption results, 94.41% at 10.202 GHz and 94.33% at 10.253 GHz for TE and TM polarizations, are similar to the ones obtained in [2]. However, the FWHM bandwidth clearly outperforms the one obtained in [2]: 663 MHz (6.5%) for TE and 675 MHz (6.58%) for TM compared to 5.82% [2] under normal incidence. In addition, the presented MMA is completely stable in both polarization and oblique incidence, whereas in [2], slight frequency shifts can be observed for some polarization angles, and the TM case is not presented under oblique incident. Furthermore, the thickness of the scaled MMA is 0.7 mm (λ/42 at its resonance frequency) compared to 1 mm (λ/29.1 at its resonance frequency) used in [2], which confirms that the proposed MMA design is thinner and, so, provides better miniaturization. The methodology proposed in [2] for bandwidth enhancement (based on coupling the resonances of several scaled unit-cells) can be applied to the unit-cell proposed in this document, and so, better results would be obtained. The same top metallization as in [2] is used in [5], where the MMA reaches an absorption peak of 96.7% at 9.92 GHz.

The MMA presented in [17] achieves an absorption peak of 98% at 10.05 GHz with an FWHM of 4.8%. Moreover, as the incident angle increases, the absorption peaks decrease, and the shifts in the resonance frequency become greater, mainly for TM polarization.

The work in [22] shows an interesting broadband MMA design. However, the broad bandwidth is obtained at a higher frequency (the bandwidth increases with resonance frequency) by using a lower relative dielectric substrate (ε_r_ = 2.66) (which also increases the bandwidth) and the same loss tangent as in this work. The main drawback is that the design is not completely angularly stable (just till 30°). The metallic ink has higher resistivity than copper, so that the absorption is improved due to ohmic losses. Nevertheless, the manufacturing technique is very promising.

The design presented in [23] is a broad bandwidth absorber. However, this behavior is achieved by heightening the dielectric thickness to 3.2 mm (λ/15 at the middle of its resonance frequency). Furthermore, the design is not polarization insensitive, and the absorption and bandwidth decrease with the increase of the incident angle.

Finally, the closer MMA to the one presented here in terms of resonance frequency is described in [8]. A 99.8% absorption peak at 5.57 GHz is achieved. The bandwidth at FWHM is 220 MHz (3.95%), which is 1.07% lower than the absorption presented in this paper. In addition, the angular stability, for both TE and TM polarizations, undergoes greater resonance frequency shifts and exhibits lower absorption peaks as the incident angle increases than the star design presented in this contribution.

In sum, most of the microwave MMA exhibit similar to or smaller bandwidth as the star design but at almost double the resonance frequency (which is easier), with none having better angular stability. Additionally, the one at a similar resonance frequency shows a narrower bandwidth, a higher frequency shift and worse angular stability than the star design presented in this work.

This work also aims to achieve a more flexible MMA, so that it could be object shape adapted to some extent. For this aim, it is necessary to adjust the presented design to be fabricated in a more flexible dielectric substrate. Table 2 shows the dielectric properties of the considered substrates. It is important to point out that, even if these materials are not generally considered as flexible, their flexibility is much greater than the FR4 one, and they can be considered conformable, at least for small thicknesses.

**Table 2:** Flexible dielectrics tested.

Dielectric	ε_r_	tanδ	Thickness (mm)
Arlon25N	3.28	0.0025	0.4572
RO3003	3	0.0013	0.8
RO4003C	3.38	0.0027	0.2032
RO4003C	3.38	0.0027	0.406

Once the star design is analyzed with all of the proposed dielectrics and several thicknesses, better results concerning absorption and angular stability are obtained for Arlon25N with only one 0.457 mm-thick layer (λ_0_/109.5 at f_r_) and a 18-μm copper thickness.

**Figure 10:**
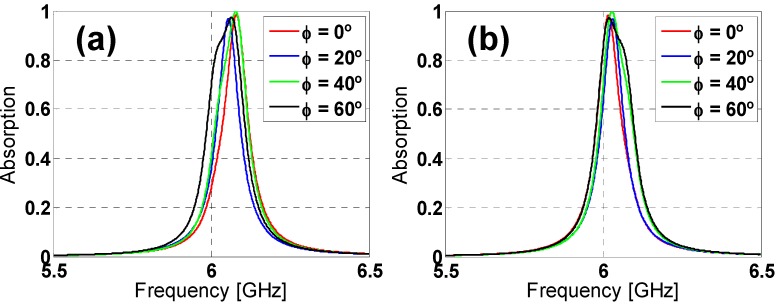
Star design absorption in simulation with Arlon25N and different polarization angles (ϕ) of the incident field for both (**a**) TE and (**b**) TM polarizations.

**Figure 11:**
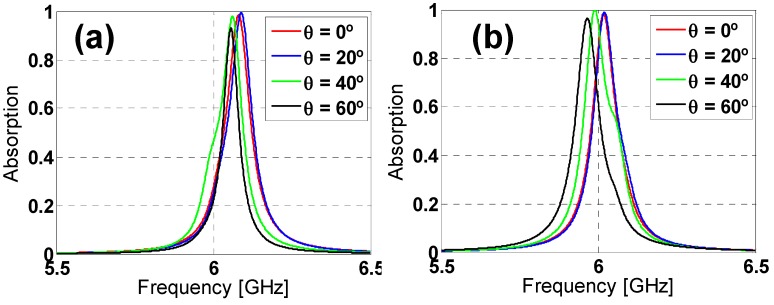
Star design absorption in simulation with Arlon25N and different incident angles (θ) for both (**a**) TE and (**b**) TM polarizations.

From Figure 10, for ϕ = 0°, nearly 100% absorption is obtained for both TE and TM polarizations using Arlon25N; more specifically: 98.37% at 6.081 GHz for TE and 98.23% at 6.018 GHz for TM. The bandwidth is 1.53% for both configurations. It could seem a narrow bandwidth, but it is necessary to take into account that the thickness is roughly half of the considered one with FR4 and preserves the same geometry of the former one, since it is not optimized for that new dielectric. Regarding the angular stability (see Figure 10 and Figure 11), it can be concluded that the design is completely angularly stable, even if there is slight shift in frequency for TM polarization under oblique incidence, due to the lower dielectric permittivity of the ARLON25N compared to the FR4 (since higher ε_r_ improves the angular stability).

### 2.2. Metamaterial Absorber Characterization

**Figure 12:**
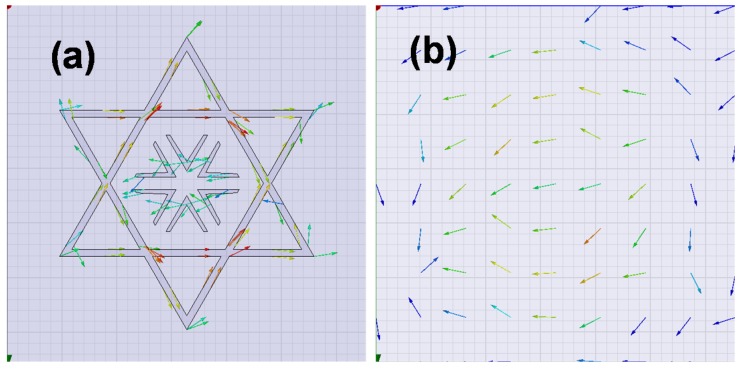
Currents on the metallization sheets: (**a**) top sheet and (**b**) bottom sheet.

**Figure 13:**
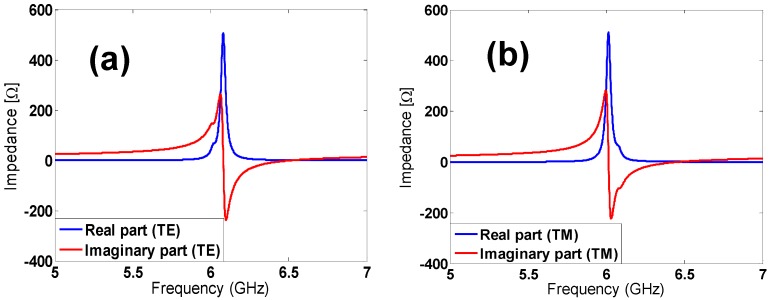
Real and imaginary part of the impedance for both the (**a**) TE configuration and (**b**) TM configuration, under normal incidence.

A study of the current distribution is carried out to better understand the magnetic response, which is due to the distribution of the surface current in both the top and bottom metamaterial unit-cell, which are clearly in opposite directions (anti-parallel currents; see Figure 12). These currents produce a magnetic field, which is opposite of the incidence one. The impedance curves are also depicted in Figure 13 and show the clear matching at the resonance frequency for both TE and TM polarizations (*Re*(*Z*) *=* 120*π* and *Im*(*Z*) *=* 0).

Once the metamaterial performance is clarified, a simplified equivalent circuit is devised to better understand the structure behavior based on a deep study of previously presented works regarding the modeling of periodic structures [24,25,26,27,28,29,30,31,32,33,34].

These kinds of metamaterials can be modeled as a grid impedance (Z_g_) parallel to a dielectric impedance (Z_d_) [7,15,16,20]. The first impedance, the grid one, is the result of the top metallization impedance and the capacitance between neighbor unit-cells; the other one is owed to the dielectric backed with the metallic plate, which is merely inductive, as was proven in [20].

**Figure 14:**
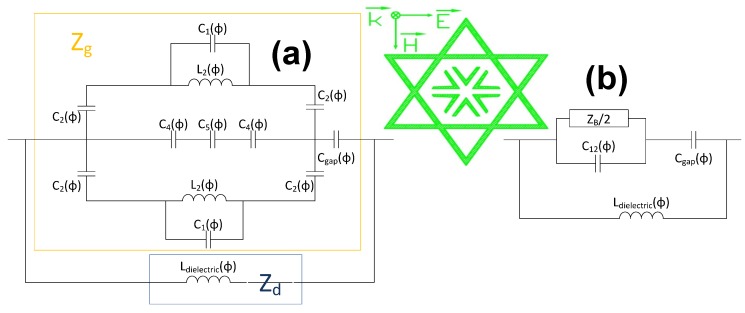
(**a**) Equivalent circuit for TE polarization and (**b**) its simplification.

**Figure 15:**
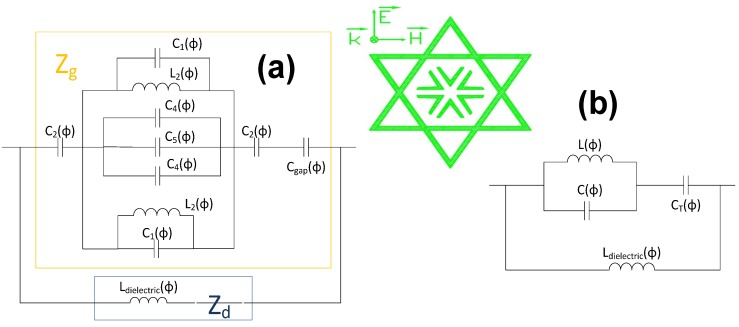
(**a**) Equivalent circuit for TM polarization and (**b**) its simplification.

The estimated circuit and its simplification are shown for both TE (Figure 14) and TM (Figure 15) polarizations. The TE circuit consists of an inductance (L_2_) parallel to a capacitor (C_1_), which are owed to the top sharped end of the star. In the middle of the structure, there are three capacitors (C_4_, C_5_ and C_4_) corresponding to the Λ-shaped metallic strips. The capacitors (C_2_) result from the side points of the star. It is noticed that identical inductances and capacitors correspond to the bottom side of the star, since this is a symmetrical structure.

The TM circuit comprises an inductance (L_2_) parallel to a capacitor (C_1_) owed to the side part of the star. The internal metallic strips can be modelled as three parallel conductors (C_4_, C_5_ and C_4_). There is a capacitor (C_1_), on the top and bottom sides, which correspond to the lower and upper star tips. The capacitor C_gap_ and the inductance L_dielectric_ are the capacitance between neighbor unit-cells and the inductance of the dielectric with the ground plane, respectively.

These structures, as stated earlier, exhibit an LC parallel-resonant behavior with the quality factor shown in Equation (2). Therefore, it can be concluded that the greater the inductive performance, the wider the bandwidth.

(2) $$Q=w_{0}C=\frac{1}{w_{0}L}=\frac{f_{0}}{BW}$$

## 3. Experimental Section

**Figure 16:**
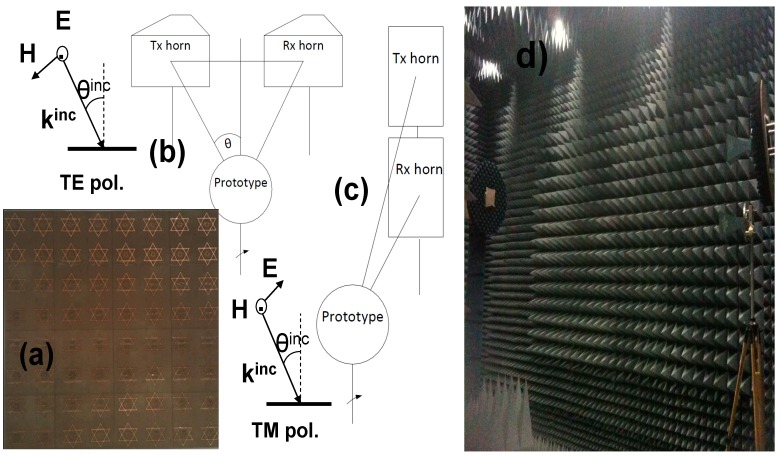
Manufactured prototype (**a**); measurement setup configurations: (**b**) TE, (**c**) TM and (**d**) measurement setup in an anechoic chamber.

An 8 × 8 unit-cell prototype has been fabricated using laser micromachining. Measurements of the reflection coefficient have been carried out in an anechoic chamber under different polarization and incidence angles taking as a reference a metallic plate [35]. A 3-meter distance is fixed between the horn antennas and the prototype to ensure that the obtained measurements are under far-field conditions. The manufactured prototype and the arrangement of the anechoic chamber are shown in Figure 16.

**Figure 17:**
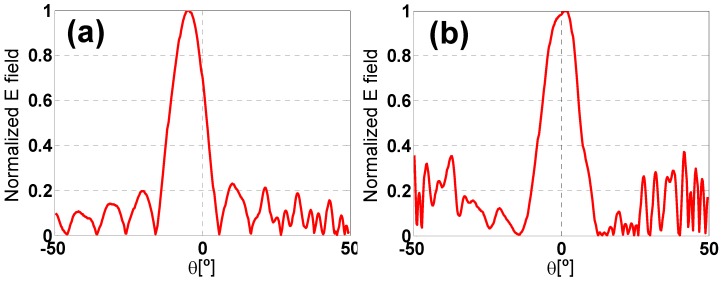
Scattered field pattern in an anechoic chamber: (**a**) TE and (**b**) TM.

The measurement of the MMA stability under different incident angles is limited by the finite size of the prototype, which determines the scattering field pattern (see Figure 17). The smaller the prototype size, the wider the main lobe and the closer the far-field distance, but the greater the differences in resonance frequency and behavior compared to the infinite-sized simulated metamaterial. For that reason, it is necessary to adopt a trade-off solution among the size of the prototype, the distance between the prototype and the antennas to meet far-field conditions and the oblique incidence angular range to be measured. This is a key aspect to consider, since it is unfortunately quite common to find in the literature measurements in which far-field conditions are not met, taking into account the size of the prototype and the distance between the prototype and antennas, but these results are compared with simulations corresponding to the incidence of a plane wave, without any justification. To our knowledge, a correspondence between measurements in the near-field and far-field in this case is not yet theoretically justified for this type of problem. Additionally, the incident plane-wave simulation requires a far-field condition. The far-field measurement of a metallic plate’s scattered field with the same size as the absorbent prototype provides the angular pattern regarding the position of the maxima and minima. Therefore, in those angular positions where the field value is very small (which can be considered as null or minima), it is not possible to calculate the angular stability. That is why many results presented using set-ups similar to the one presented here, in an anechoic chamber at microwave frequencies, are, in our humble opinion, not very believable.

**Figure 18:**
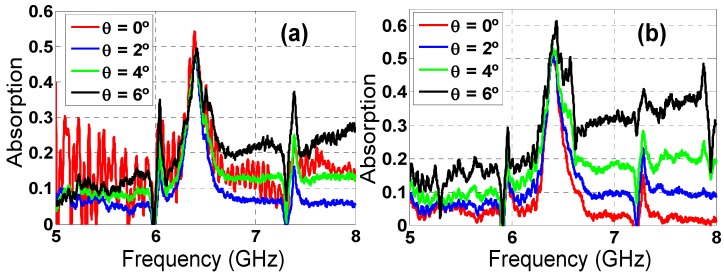
Star prototype measured absorption for different incident angles (θ) for both (**a**) TE and (**b**) TM polarizations.

According to Figure 18, an angular margin of θ = ±6° can be measured for ϕ = 0° at f_r_ = 6.394 GHz, but it is also possible to obtain measurement results for an angle of θ = 15° and ϕ = 0°, since there is a secondary lobe in the scattered field pattern and there is enough dynamic range to obtain a clear absorption peak. There is a null in the scattered field pattern for angles from θ = 8° to θ = 10°, which prevents performing a proper measurement. Even though the scattered field pattern is different for TE and TM configurations, the secondary lobes and the first null are roughly in the same place. The resonance frequency shift compared to simulation is attributable to manufacturing tolerances, finite prototype dimensions, diffraction and dispersion on the edges. The difference between the absorption measured and the one obtained in the simulation is mainly due to the set-up. Therefore, the incident field is not completely coupled in the prototype. For that reason, the field not coupled is scattered, and it is not absorbed. Another source of error is the slight differences in the placement of the prototype and the calibrating metallic plate, which lead to errors in the calibration and, hence, in the calculation of the absorption. These results are consistent with the ones presented in the literature [36]. Nonetheless further work concerning improvement on the measurement setup capabilities is being performed by the authors along with the consideration of other characterization methods.

## 4. Conclusions

A novel MMA has been designed and characterized. The significant achievements concerning both angular stability and bandwidth of this simple unit-cell have been presented. A parametric unit-cell study has been conducted, so that the resonance frequency can be easily adjusted.

Moreover, a thinner and even smaller MMA has been designed while preserving proper bandwidth and angular stability performance. The measurement results of the fabricated prototype agreed fairly well with the simulation ones considering the challenges in the measurement process.

Finally, an extension to enlarge the bandwidth through coupling several resonances (either scaling the unit-cell or adding several layers) is left for future works.
